# Hybrid Biostimulator and Hyaluronic Acid Injectable for Tear Trough Rejuvenation

**DOI:** 10.1007/s00266-026-05817-z

**Published:** 2026-05-07

**Authors:** Stephanie Ming Young, Edmond K. H. Lau

**Affiliations:** 1 Eagle Aesthetics and Surgery, King Albert Park, Singapore; 2 National University Hospital, Kent Ridge, Singapore; 3 EC Skin & Laser Clinic, Penang, Malaysia

**Keywords:** Tear trough correction, PDLLA, Biostimulator injection, Periorbital rejuvenation

## Abstract

**Purpose:**

Non-surgical correction of the under eye region is challenging due to various reasons. Our study evaluates a novel biostimulator poly-D,L-lactic acid–hyaluronic acid (PDLLA-HA) 50mg (Juvelook, VAIM Inc., Seoul, Republic of Korea), a poly-D,L-lactic acid product as a potential injectable for tear trough correction.

**Methods:**

This is a prospective cohort study evaluating patients who underwent three injections of PDLLA-HA for correction of the tear trough and under eye region. Outcome measures included: Perceived improvement by investigators and patients using a 5-grade Global Aesthetic Improvement Scale (GAIS), skin features assessment by investigators using a 5-point subjective scale based on four criteria (elasticity, hydration, skin quality and radiance), patient satisfaction and adverse events (if any).

**Results:**

Our study comprised 15 females, with a mean age of 38.5 years. Tear trough grading ranged from 1 to 3, with a mean grading of 1.93. Mean PDLLA-HA used: 0.41 (RE), 0.41 (LE) at first visit; 0.45 (RE), 0.45 (LE) at second visit; and 0.45 (RE), 0.45 (LE) at third visit. Mean GAIS for tear trough for investigator improved from 2.93 to 2.2 to 2.13, and for patients from 2.73 to 1.93 to 1.8 after first treatment, second treatment and third treatment, respectively. There was significant improvement in scores for lower lid skin elasticity, hydration, quality and radiance (p<0.001, Friedman). All patients were keen to repeat the treatment when asked at the second, third and fourth visits. There were no adverse events reported.

**Conclusion:**

PDLLA-HA is a potential injectable for tear trough correction as well as for improvement in under eye skin elasticity, hydration, quality and radiance.

**Level of Evidence IV:**

This journal requires that authors assign a level of evidence to each article. For a full description of these Evidence-Based Medicine ratings, please refer to the Table of Contents or the online Instructions to Authors www.springer.com/00266.

**Supplementary Information:**

The online version contains supplementary material available at 10.1007/s00266-026-05817-z.

## Introduction

The tear trough is the natural depression extending inferolaterally from the medial canthus [1]. This natural anatomical structure becomes accentuated with age due to changes in the skin and periorbital region, and may be called a “tear trough deformity” [2].

Skin and fat atrophy as well as attenuation of the periorbital structures allow herniation of orbital fat and an increase in shadowing [3]. The tear trough and lid–cheek junction are further accentuated by overlying pigmentation, skin texture and shadow changes. This is a significant concern to patients, who often report a “tired” or “aged” under eye appearance that is not relieved with rest, hydration or topical therapies [4].

In recent years, there has been considerable interest in non-surgical treatment of the tear trough using injectable products such as hyaluronic acid (HA) to temporarily fill the depression [5]. With the proper placement of injectables, effective rejuvenation of the periorbital region by volumizing and restoring support to the infraorbital hollows is possible in some cases [4]. However, this region has several anatomical features that are challenging for the injector. First, the skin is extremely thin and translucent, and depending on the filler injected, there is a risk of visible lumps or the Tyndall effect, a bluish discoloration due to the optical properties of the filler used. The vascularity of the tear trough area means that the physician must inject carefully or risk bleeding and bruising. The area is also very susceptible to oedema because of compromised lymphatic drainage in the superficial suborbicularis oculi fat compartment [6].

While there has been a rise in the use of biostimulator injectables in the aesthetic industry [7, 8], these injectables are generally not actively promoted as under eye rejuvenation due to their properties and potential risk of nodules and granulomas, especially in the thin under eye area.

Our study evaluates the efficacy and safety of a novel injectable for tear trough and under eye rejuvenation in an Asian population: PDLLA-HA 50mg (Juvelook, VAIM Inc., Seoul, Republic of Korea), a poly-D,L-lactic acid (PDLLA) product containing 42.5mg of spongiform spherical PDLLA polymer with 7.5mg of non-cross-linked hyaluronic acid (HA). It is a hybrid skin booster/collagen stimulator injectable that has several studies showing its safety and collagen stimulation benefits [9–13]. Its properties and particle size also make it a potential injectable for the under eye region [14, 15].

## Methods

### Inclusion and Exclusion Criteria

We carried out a prospective cohort study at two independent clinics serving an East Asian and South Asian population from September 1, 2024, to September 30, 2025. Two doctors were involved in the study: Dr SY from Eagle Aesthetics and Surgery, Singapore, and Dr EL from EC Clinic, Penang, Malaysia. Inclusion criteria were as follows: any subject with tear trough deformity between Class I to III according to Hirmand classification (Class I patients have volume loss limited medially to the tear trough and can also have mild flattening extending to the central cheek; Class II patients exhibit volume loss in the lateral orbital area in addition to the medial orbit and may have moderate volume deficiency in the medial cheek and flattening of the central upper cheek; and Class III patients present with a full depression circumferentially along the orbital rim, medial to lateral), above 18 years of age and capable of giving consent, and able to complete three Juvelook sessions to the under eye region with at least 3 months follow-up after the third Juvelook session. We excluded patients with significant eyelid laxity, significant orbital fat prolapse and patients with prior injection of permanent filler in this region. The study was approved by the Parkway Independent Ethics Committee (PIEC) and adhered to the tenets of the Declaration of Helsinki. Informed consent was obtained from all patients in the study, including consent to publish identifiable photographs.

### Injection Technique

**Fig. 1:**
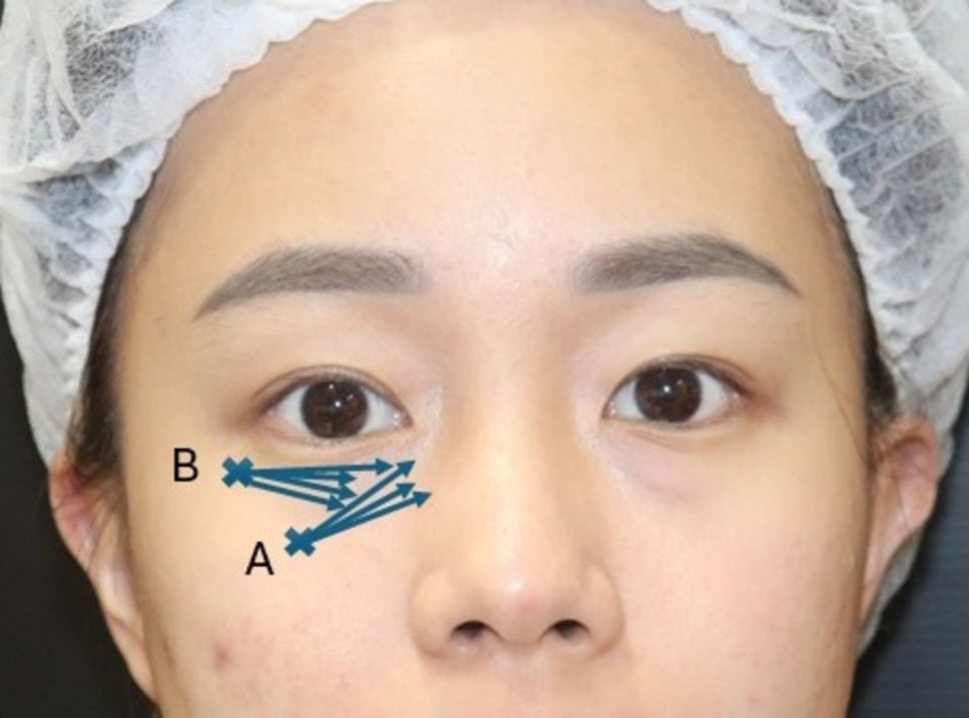
See Video technique for under eye PDLLA-HA injection. Using a 25G or 27 G cannula, the PDLLA-HA injection was delivered via two entry points—the first entry point (A) was approximately at level of orbital rim just lateral to landmark of infraorbital foramen, while the second entry point was just inferior to lateral canthus (B). Through the first entry point (A), PDLLA-HA was injected into the tear trough deformity. The deepest portion of the medial tear trough was treated first. The cannula was then threaded and PDLLA-HA injected subdermally above the orbicularis oculi in a linear retrograde technique, with care taken to avoid going posterior to septum. Approximately 0.1–0.3ml of PDLLA-HA was injected into tear trough deformity for each lower eyelid. Next, the cannula was introduced through the second injection point (B) and delivered to the lateral and central aspect of the lower eyelid that may not have been covered by the initial injection through the first entry point targeting the tear trough. A range of 0.1-0.3ml was used for this second entry point. A total volume of 0.3-0.5ml PDLLA-HA was used for each lower eyelid. Gentle massage of the PDLLA-HA was performed after procedure to even out the product

PDLLA-HA 50mg was prehydrated with 6ml normal saline for at least 24 hours prior. The vial of PDLLA-HA 50mg was mixed in Vortex mixer 30 minutes before injection. 1ml of PDLLA-HA was drawn into 1cc syringe. A 25 gauge (G) or 27 G cannula was used. The PDLLA-HA injection was delivered via two entry points—the first entry point was approximately at level of orbital rim just lateral to landmark of infraorbital foramen, while the second entry point was just inferior to lateral canthus (see Fig. 1). Through the first entry point, PDLLA-HA was injected into the tear trough deformity (See Video). The deepest portion of the medial tear trough was treated first. The cannula was then threaded and PDLLA-HA injected subdermally above the orbicularis oculi in a linear retrograde technique. Caution was taken to avoid going posterior to septum. Approximately 0.1-0.3ml of PDLLA-HA was injected into tear trough deformity for each lower eyelid. Next, the cannula was introduced through the second injection point and delivered to the lateral and central aspect of the lower eyelid that may not have been covered by the initial injection through the first entry point targeting the tear trough. A range of 0.1–0.3ml was used for this second entry point. A total volume of 0.3-0.5ml PDLLA-HA was used for each lower eyelid. Gentle massage of the PDLLA-HA was performed after procedure to even out the product.

The above was repeated monthly, for a total of three times, as part of the recommended “boost” protocol for all patients undergoing the injection for the first time. The patients were then recommended a maintenance protocol of 6 monthly injections, or as required.

### Outcome Measures

Perceived improvement was assessed by the investigators and the patients using a 5-grade Global Aesthetic Improvement Scale (GAIS) (Table 1 and Data Collection Sheet). Skin features were also assessed by the investigators before injection and at the follow-up visit using a 5-point subjective scale based on four criteria (elasticity, hydration, skin quality and radiance) (Table 1 and Data Collection Sheet). Patients were also directly asked whether they would agree to repeat the same treatment and would recommend the treatment to relatives. The patients’ satisfaction forms were filled in contemporaneously. The volume of PDLLA-HA injected per side was recorded. Safety was evaluated by reporting all adverse events and injection site reactions, at each visit, by the investigators. Data were collected at one month after the first injection, one month after the second injection and one month after the third injection.

**Table 1:** Global aesthetic improvement scale and skin grading

Global aesthetic improvement scale (GAIS)		
	Degree	Description
1	Exceptional improvement	Excellent corrective result
2	Very improved patient	Marked improvement of the appearance, but not completely optimal
3	Improved patient	Improvement of the appearance, better compared with the initial condition, but a touch-up is advised
4	Unaltered patient	The appearance substantially remains the same compared with the original condition
5	Worsened patient	The appearance has worsened compared with the original condition

Skin assessment: elasticity, hydration, skin quality and radiance	
1	Very satisfactory
2	Satisfactory
3	Moderately satisfactory
4	Unsatisfactory
5	Very unsatisfactory

## Results

Our study comprised 15 females, with a mean age of 38.5 years (age range 24-51 years). The tear trough grading ranged from 1 to 3, with a mean grading of 1.93. Mean PDLLA-HA used was as follows: 0.41ml (RE), 0.41ml (LE) at first visit; 0.45ml (RE), 0.45ml (LE) at second visit; and 0.45ml (RE), 0.45ml (LE) at third visit. The volume for each under eye ranged from 0.3ml to 0.5ml at each visit.

**Fig. 2:**
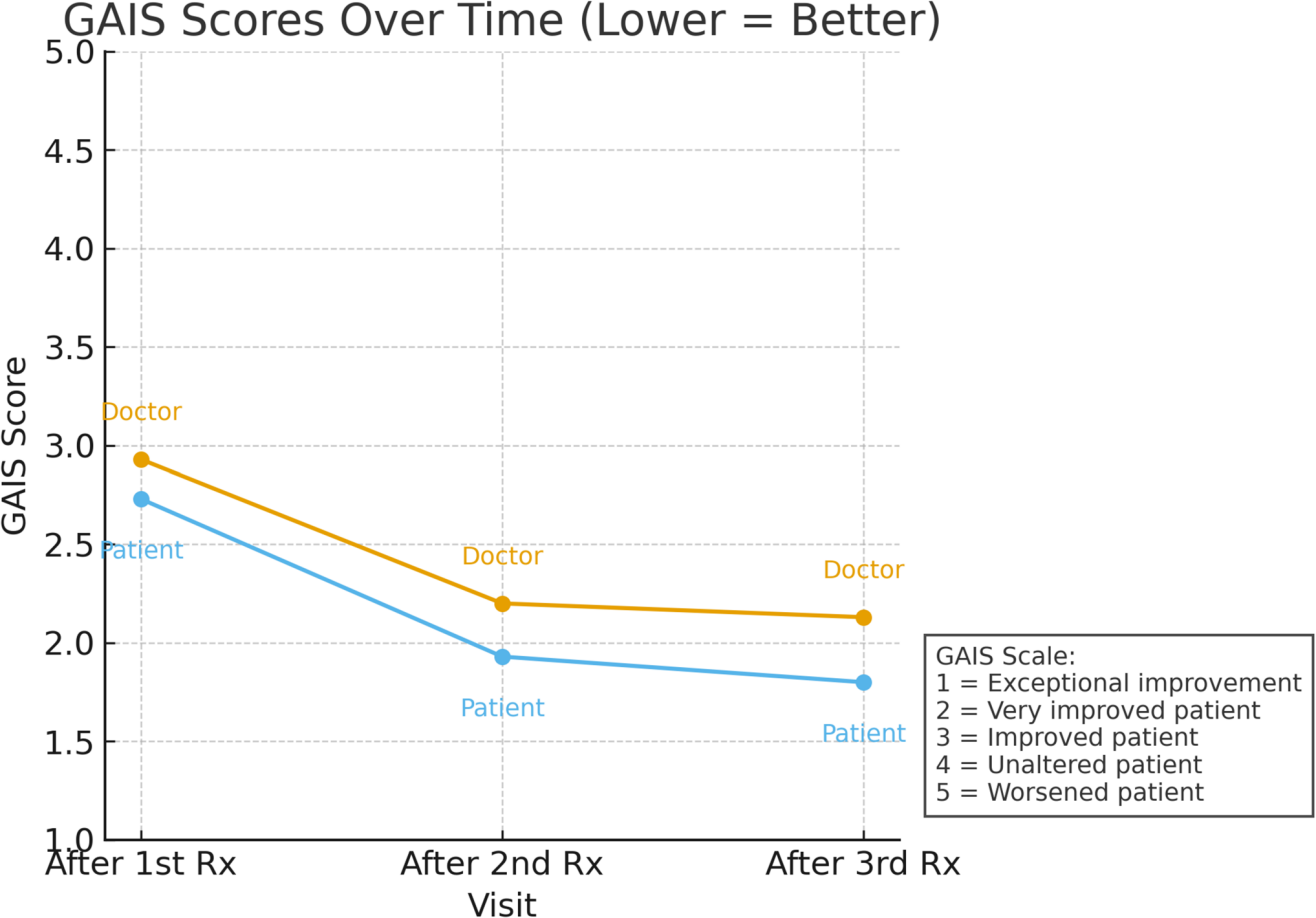
Global Aesthetic Improvement Scale Improvement (GAIS). Mean GAIS for tear trough for doctors improved from 2.93 to 2.2 to 2.13, and for patients from 2.73 to 1.93 to 1.8 after first treatment, second treatment and third treatment, respectively

**Fig. 3:**
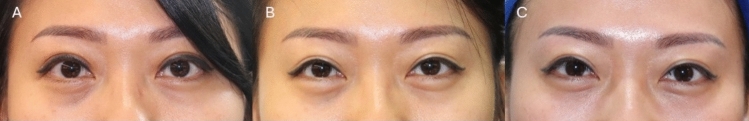
Clinical improvement with PDLLA-HA to under eye. Patient had tear trough grade 2 (Hirmand classification) (**A**) which improved to grade 1 after one PDLLA-HA injection (**B**). She had further improvement to her tear trough and overall skin quality, radiance and hydration after three PDLLA-HA injections (**C**)

**Fig. 4:**
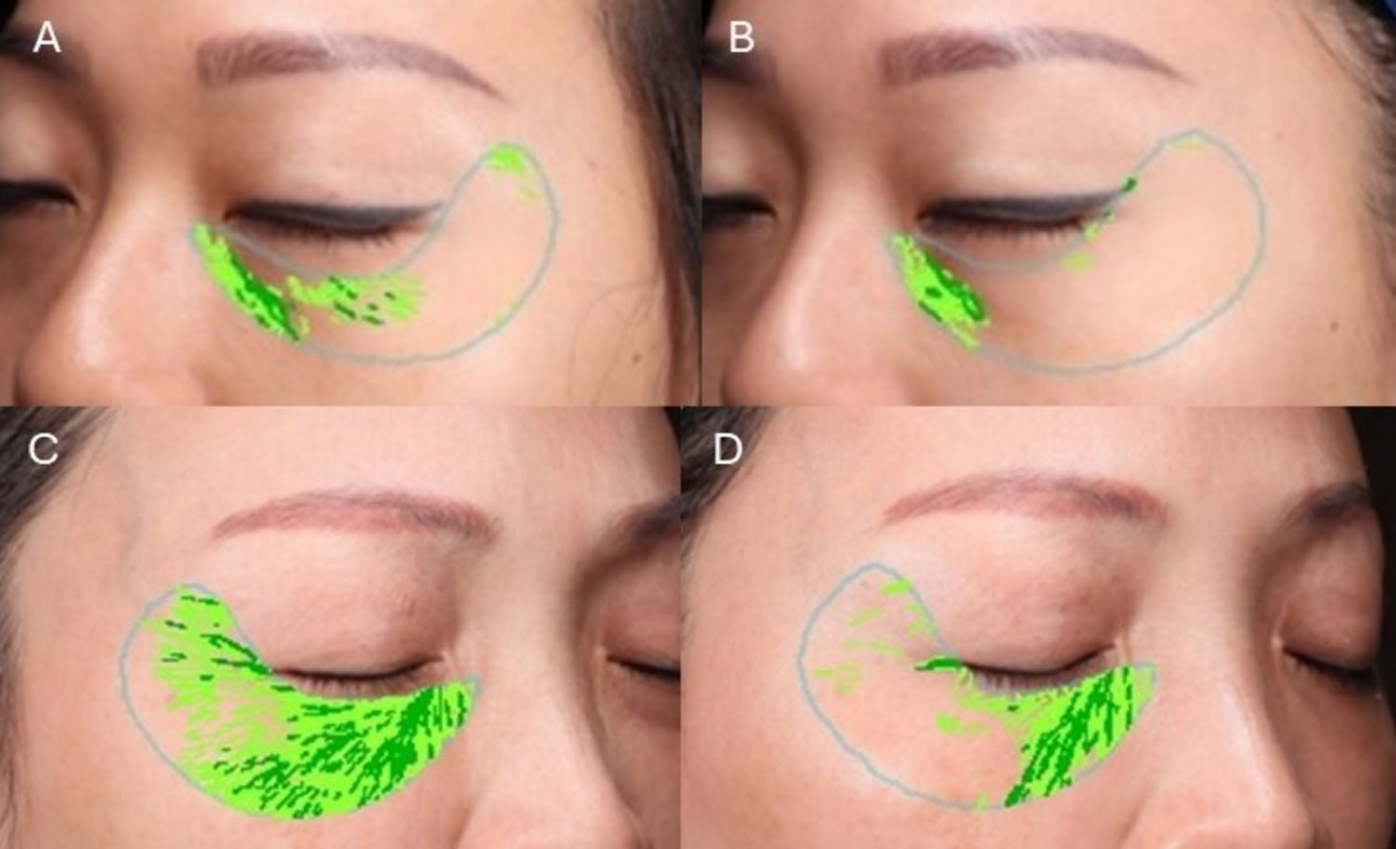
Clinical improvement with PDLLA-HA injection to under eye. (**A**) Patient had tear trough grade 3 (Hirmand classification) with some fat protrusion. This improved to grade 2 after three PDLLA-HA injections with improvement in skin quality (**B**). Patient showed improvement in periorbital fine lines and wrinkles from before (**C**) to after (**D**) the injections

Figure 2 shows the improvement in GAIS for tear trough for both investigator and patients, with mean GAIS for investigator (GAISi) improving from 2.93 (after first treatment) to 2.2 (after second treatment) to 2.13 (after third treatment), and mean GAIS for patients (GAISp) improving from 2.73 (after first treatment) to 1.93 (after second treatment) to 1.8 (after third treatment). Figures 3 and 4 demonstrate the clinical improvement seen in the tear trough and under eye region.

**Fig. 5:**
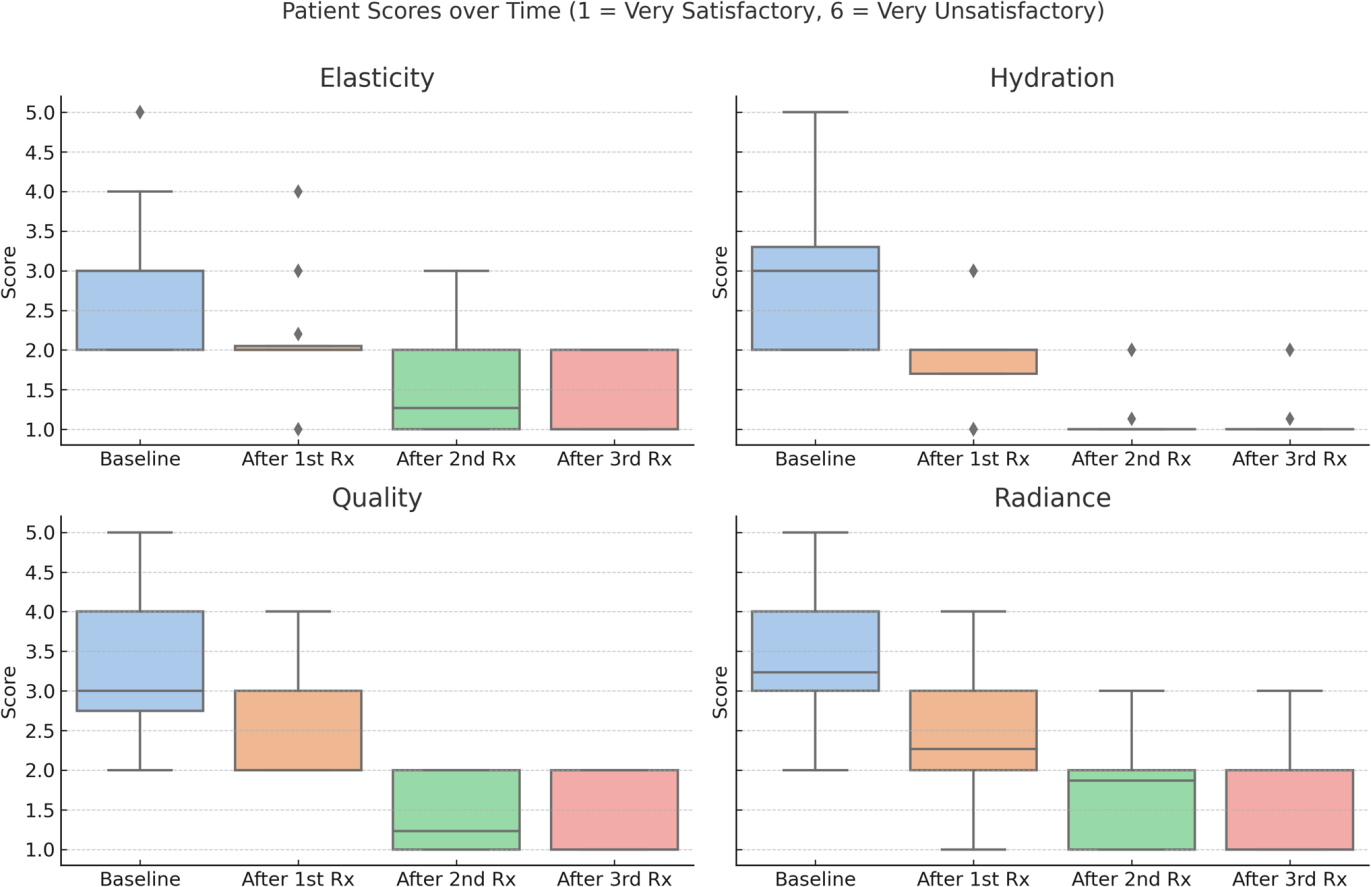
Scores for lower lid elasticity, hydration, quality and radiance. Skin features assessed by investigators before injection and at the follow-up visit using a 5-point subjective scale based on four criteria (elasticity, hydration, skin quality and radiance) showed improvement in scores as follows: Skin elasticity improved from 2.93 to 2.2 to 1.53 to 1.4; skin hydration from 3.06 to 1.93 to 1.13 to 1.13; skin quality from 3.2 to 2.4 to 1.46 to 1.13; and skin radiance from 3.46 to 2.53 to 1.73 to 1.6 from first visit to after first treatment to after second treatment to after third treatment, respectively

Figure 5 shows significant improvement in scores for lower lid skin elasticity, hydration, quality and radiance (p<0.001, Friedman). Skin elasticity improved from 2.93 to 2.2 (after first treatment) to 1.53 (after second treatment) to 1.4 (after third treatment). Skin hydration scores improved from 3.06 to 1.93 (after first treatment) to 1.13 (after second treatment) and remained at 1.13 (after third treatment). Skin quality improved from 3.2 to 2.4 (after first treatment) to 1.46 (after second treatment) to 1.13 (after third treatment). Skin radiance improved from 3.46 to 2.53 (after first treatment) to 1.73 (after second treatment) to 1.6 (after third treatment).

Mean patient satisfaction was 2.6 (after first treatment), 1.73 (after second treatment) and 1.73 (after third treatment). All patients (100%) said they would repeat the treatment when asked at the second, third and fourth visits. Most of the patients had mild swelling in the under eye region for a few hours to two days, which resolved spontaneously. There were temporary bruises in two patients at the injection sites (which self-resolved in a few days) with no other adverse events reported.

## Discussion

Our study shows PDLLA-HA (Juvelook) to be a potential injectable for the under eye area, with improvement in tear trough appearance as well as under eye skin elasticity, hydration, quality and radiance. The greatest improvement in GAIS was observed between the first and second treatments, with subsequent changes showing a more gradual trend. Patient satisfaction was good with all patients reporting a willingness to repeat the treatment. No adverse events were encountered in our study.

Anatomical features explaining the tear trough are as follows: 1) The skin is thinner over the palpebral orbicularis oculi and thicker over the orbital orbicularis oculi; 2) immediately deep to skin, there is no subcutaneous fat over the palpebral orbicularis oculi, with the tear trough corresponding to the junction of the palpebral (preseptal) and orbital portions of the orbicularis oculi muscle; 3) in the suborbicularis plane, the tear trough corresponds to the origin of the palpebral portion of the orbicularis oculi from the maxilla, extending obliquely from the medial canthus to a point 4–6 mm below the orbital rim; and 4) orbital fat herniation accentuates tear trough and lid/cheek junction [2].

Atrophy of the skin and underlying subcutaneous fat is the most likely explanation for the increasing visibility of the tear trough and lid/cheek junction with age. It is unlikely that there is age-related descent of these structures because they are fixed to the bone. Several procedures have been described to improve the appearance of the tear trough and lid/cheek region, including midface lifts, orbicularis redraping, orbital fat transposition and the injection of fat and hyaluronic acid fillers [2, 16].

The ideal and most physiologic treatment for these landmarks would appear to be in the superficial plane between the skin and the underlying muscle. The problem is that the skin is extremely thin and interventions in this plane may leave contour irregularities. Injection of material above the muscle is fraught with hazard. Injected fat frequently does not resorb and is difficult to remove without creating a greater deformity. Fat injections are time consuming because of the need to harvest fat, expensive because they require a sterile surgical field, and carry risks of lumpiness and uncertain volume retention over time [17, 18]. There may be a role for the injection of HA filler in this superficial plane, but there is risk of visible lumps or the Tyndall effect. The area is also very susceptible to oedema because of compromised lymphatic drainage in the superficial suborbicularis oculi fat compartment [6, 16].

For these reasons, there is a role for biostimulator injectables for improvement of the under eye skin quality, particularly above and cephalic to the tear trough region, to improve the appearance of the tear trough deformity. Our study correlates with the single other study on PDLLA-HA on under eye rejuvenation, where four patients underwent PDLLA-HA injection in the under eye area and demonstrated marked improvements after a single session of treatment with a substantial reduction in hollowness and enhanced facial aesthetics [13]. The difference between their study and ours is the volume used—our study used a more conservative volume of 0.3 to 0.5ml for each eye under eye for each treatment, while theirs used 1ml. We also evaluated our patients over three treatment sessions, while theirs evaluated patients after one treatment (albeit at a higher dose). Nonetheless, results from both studies were promising, with PDLLA-HA showing ability to address various degrees of tear trough deformities.

For non-surgical interventions in the periorbital region, a more conservative approach is preferred owing to the subtle anatomy of this region. PDLLA-HA has shown promise as an injectable for the under eye region with its dual mechanism. The HA gel carrier allows subtle yet sufficient early volumization and yet potentially avoiding the problem of swelling or Tyndall from as it is a non-cross-linked HA. Meanwhile, PDLLA has been shown to be effective in stimulating the body's own collagen production, leading to gradual and sustained improvements in skin quality and volume [10, 11]. This biological stimulation of collagen helps to rebuild and strengthen the skin's structure over time, offering longer‐lasting results with fewer maintenance sessions. Imaging studies have also demonstrated immediate volumetric gain and skin texture enhancement with PDLLA-HA [15].

To date, there is very little in the literature which can show improvement in the tear trough deformity, as well as under eye ageing, by addressing the thin skin over the palpebral orbicularis oculi. Previous attempts to “conceal” the tear trough by adding HA filler or fat to the deformity have led to less than desirable results. Perhaps with the rise in research and technology, the next wave of injectables such as PDLLA-HA will focus on improving the skin quality of the periorbital skin, to reduce the difference in skin quality between the skin over the palpebral orbicularis oculi and orbital orbicularis oculi, as well as to address the atrophy of skin and subcutaneous fat, which are important anatomical causes of the tear trough deformity.

There are limitations to our paper. It is a small uncontrolled pilot cohort study, although the largest series of PDLLA-HA 50mg for under eye rejuvenation to date. It is also limited as by the study population which is female and Asian. A larger cohort would provide more robust data, allowing for a better understanding of the range of outcomes in different patient populations. A control group to directly compare the efficacy and safety of PDLLA to other fillers would be ideal though not practically difficult. High-definition radiological and even histological studies looking at the treated under eye skin may be the next step forward. While the safety profile of PDLLA-HA appears good, longer-term studies are necessary to assess the durability of the effects and to understand the long‐term safety profile of PDLLA in the periorbital region.

In summary, our study shows PDLLA-HA to be a potential injectable for the under eye area, with improvement in tear trough appearance as well as under eye skin elasticity, hydration, quality and radiance, with good patient satisfaction and safety profile.

## Supplementary Information

Below is the link to the electronic supplementary material.

Supplementary file1 (MP4 88353 KB)

## References

[1] Flowers RS. Tear trough implants for correction of tear trough deformity. Clin Plast Surg. 1993;20:403–15. 8485949

[2] Haddock NT, Saadeh PB, Boutros S, Thorne CH. The tear trough and lid/cheek junction: anatomy and implications for surgical correction. Plast Reconstr Surg. 2009;123(4):1332–40. 19337101 10.1097/PRS.0b013e31819f2b36

[3] Lambros V. Observations on periorbital and midface aging. Plast Reconstr Surg. 2007;120(5):1367–76. 17898614 10.1097/01.prs.0000279348.09156.c3

[4] Trinh LN, Grond SE, Gupta A. Dermal fillers for tear trough rejuvenation: a systematic review. Facial Plast Surg. 2022;38(3):228–39. 34192769 10.1055/s-0041-1731348

[5] Huber-Vorländer J, Kürten M. Correction of tear trough deformity with a cohesive polydensified matrix hyaluronic acid: a case series. Plast Surg Nurs. 2015;35(4):171–6. 26605822 10.1097/PSN.0000000000000112

[6] Funt DK. Avoiding malar edema during midface/cheek augmentation with dermal fillers. J Clin Aesthet Dermatol. 2011;4:32–6. 22191006 PMC3244361

[7] Signori R, Barbosa AP, Cezar-Dos-Santos F, Carbone AC, Ventura S, Nobre BBS, et al. Efficacy and safety of Poly-l-Lactic Acid in facial aesthetics: a systematic review. Polymers (Basel). 2024;16(18):2564. 39339028 10.3390/polym16182564 PMC11435306

[8] Attenello NH, Maas CS. Injectable fillers: review of material and properties. Facial Plast Surg. 2015;31(1):29–34. 25763894 10.1055/s-0035-1544924

[9] Oh S, Seo SB, Kim G, Batsukh S, Park CH, Son KH, et al. Poly-D,L-Lactic Acid filler increases extracellular matrix by modulating macrophages and adipose-derived stem cells in aged animal skin. Antioxidants. 2023;12(6):1204. 37371934 10.3390/antiox12061204 PMC10294940

[10] Oh S, Seo SB, Kim G, Batsukh S, Son KH, Byun K. Poly-D,L-Lactic acid stimulates angiogenesis and collagen synthesis in aged animal skin. Int J Mol Sci. 2023;24(9):7986. 37175693 10.3390/ijms24097986 PMC10178436

[11] Lee KWA, Chan LKW, Lee AWK, Lee CH, Wong STH, Yi KH. Poly-d,l-lactic Acid (PDLLA) application in dermatology: a literature review. Polymers (Basel). 2024;16(18):2583. 39339047 10.3390/polym16182583 PMC11434839

[12] Seo SB, Park H, Jo JY, Ryu HJ. Skin rejuvenation effect of the combined PDLLA and non cross-linked hyaluronic acid: a preliminary study. J Cosmet Dermatol. 2024;23(3):794–802. 37969055 10.1111/jocd.16085

[13] Wan J, Hidajat IJ, Cartier H, Garson S, Bautzer C, Machado LB, et al. Evaluating poly-D,L-lactic acid for lower eyelid rejuvenation: efficacy and safety. J Cosmet Dermatol. 2025;24(3):e70058. 40017393 10.1111/jocd.70058 PMC11868823

[14] Seo SB, Wan J, Yi KH. Energy-based device management of nodular reaction following poly-D, L-lactic acid injection for tear trough rejuvenation. J Cosmet Dermatol. 2025;24(1):e16575. 39283001 10.1111/jocd.16575 PMC11743236

[15] Ravera K. Ultrasound and radiological patterns in assessing the efficacy of Juvelook for collagen stimulation and tear trough enhancement: a case report. Cureus. 2025;17(4):e82668. 40400849 10.7759/cureus.82668 PMC12093134

[16] Woodward J, Cox SE, Kato K, Urdiales-Galvez F, Boyd C, Ashourian N. Infraorbital hollow rejuvenation: considerations, complications, and the contributions of midface volumization. Aesthet Surg J Open Forum. 2023;17:5:ojad016. 10.1093/asjof/ojad016 PMC10045888 36998744

[17] Lipp M, Weiss E. Nonsurgical treatments for infraorbital rejuvenation: a review. Dermatol Surg. 2019;45(5):700–10. 31033596 10.1097/DSS.0000000000001897

[18] Stutman RL, Codner MA. Tear trough deformity: review of anatomy and treatment options. Aesthet Surg J. 2012;32(4):426–40. 22523096 10.1177/1090820X12442372

